# Range of protein induced by vitamin K absence or antagonist-II levels in neonates at birth

**DOI:** 10.1038/s41598-024-51674-8

**Published:** 2024-01-09

**Authors:** Tomohiro Sameshima, Mariko Ashina, Takuya Fukuda, Takumi Kido, Shinya Abe, Yuko Watanabe, Itsuko Sato, Yoshihiko Yano, Kenji Tanimura, Hiroaki Nagase, Kandai Nozu, Kazumichi Fujioka

**Affiliations:** 1 Department of Pediatrics, Kobe University Graduate School of Medicine, 7-5-2, Kusunoki-cho, Chuo-ku, Kobe, 650-0017 Japan; 2 Department of Clinical Laboratory, Kobe University Hospital, Kobe, Japan; 3 Department of Obstetrics and Gynecology, Kobe University Graduate School of Medicine, Kobe, Japan

**Keywords:** Biomarkers, Diagnostic markers, Biomarkers, Paediatric research

## Abstract

Protein induced by vitamin K absence or antagonist-II (PIVKA-II) is avitamin K (VK) deficiency indicator in neonates. However, PIVKA-II detection frequency in neonatal blood at birth and the correlation between PIVKA-II and gestational age are unclear. We retrospectively analyzed infants admitted to our institution between June 1, 2018, and March 31, 2022, whose clinical and PIVKA-II data were available, and classified them into preterm and term infant groups. Overall incidence of PIVKA-II-positive cases (≥ 50 mAU/mL) was 42.8%, including 0.6% apparent VK deficiency (≥ 5000 mAU/mL), 3.1% experimental VK deficiency (1000–4999 mAU/mL), and 10.7% latent VK deficiency (200–999 mAU/mL) cases. Incidence of PIVKA-II-positive cases was significantly higher in the term group than in the preterm group (49.4% vs. 29.7%, *p* < 0.001). Gestational age correlated with PIVKA-II levels (r^2^ = 0.117, *p* < 0.0001). Median serum PIVKA-II levels and incidence of PIVKA-II-positive cases (≥ 50 mAU/mL, 16.4%) were lower at 5 days after birth than at birth, possibly reflecting the postnatal VK prophylaxis impact. Only one infant was diagnosed with VK deficiency bleeding (PIVKA-II levels, at birth: 10,567 mAU/mL; at day 5: 2418 mAU/mL). Thus, serum PIVKA-II levels after birth weakly correlated with gestational age. VK deficiency was more common in term infants than in preterm infants.

## Introduction

Neonates are physiologically prone to vitamin K (VK) deficiency. Protein induced by vitamin K absence or antagonist-II (PIVKA-II) is a functionally abnormal prothrombin that is widely used as an indicator of VK deficiency in neonates^1^. However, there are no established criteria regarding abnormal PIVKA-II levels to prevent bleeding due to VK deficiency in neonates. In adults, a PIVKA-II level ≥ 5000 mAU/mL is defined as apparent VK deficiency, considering the range observed in stable patients on warfarin therapy (aiming for prothrombin time-international normalized ratio ≥ 1.5) of 6.9–99.5 AU/mL^2,3^. In laboratory studies, a PIVKA-II level ≥ 1000 mAU/mL represents an unequivocally abnormal undercarboxylated level of prothrombin, indicating experimental VK deficiency^3,4^. However, a report has defined a PIVKA-II level ≥ 200 mAU/mL as the cutoff for defining latent VK deficiency in neonates^5^. This is because the enzyme-linked immunosorbent assay (ELISA) previously used to measure PIVKA-II levels had a minimum sensitivity of 200 mAU/mL^6,7^. Currently, chemiluminescent enzyme immunoassay (CLEIA) is mainly used to measure PIVKA-II; this assay has a higher sensitivity of ≥ 1 mAU/mL^8^. Using this method, a previous study proposed ≥ 50 mAU/mL as a cutoff for screening hepatocellular carcinoma development in high-risk adult patients^9^.

To date, various studies have measured PIVKA-II in cord blood to determine the VK deficiency status at birth in neonates. In 1986, Shapiro et al. measured PIVKA-II levels in the cord blood of 934 neonates using a modified Ap-latex method^10^. They reported that PIVKA-II was detectable in 2.9% of them. They also observed that all PIVKA-II-positive cases were term infants with gestational ages 37–42 weeks^11^. In 1993, Bovill et al. measured PIVKA-II in the cord blood of 397 newborns (including 76 preterm infants with gestational ages < 34 weeks) using their original immunoassay and reported that 8.1% of infants had detectable PIVKA-II levels^12^. In recent years, PIVKA-II in cord blood has been measured using ELISA. It was reported that 47% of term infants in the United States^7^, 16% of 683 infants (including 16 preterm infants) in Thailand^3^, and 66% of 141 infants (including 13 preterm infants) in Uganda^13^ had detectable PIVKA-II levels (≥ 100 mAU/mL, > 150 mAU/mL, and ≥ 200 mAU/mL, respectively). The only study that measured PIVKA-II in cord blood using CLEIA included 75 preterm infants in India; PIVKA-II was detected in 49% of these infants (> 28 mAU/mL)^14^. Although it is widely known that cord blood can be used as a substitute for neonatal blood, it is not possible to collect a sufficient amount of cord blood in all cases^15^. Therefore, we believe that data on the range of PIVKA-II in neonatal blood at birth are more clinically useful. However, to date, no large-scale studies involving preterm infants have performed highly sensitive PIVKA-II measurements using neonatal blood.

This study aimed to clarify the frequency of PIVKA-II detection in neonatal blood immediately after birth and examine the correlation between PIVKA-II and gestational age.

## Results

### Characteristics of the participants

A total of 2042 newborns were admitted to our institution between June 1, 2018, and March 31, 2022. Of these infants, 283 were excluded because of a lack of serum PIVKA-II data at birth. All the required data were available for the remaining 1759 infants. The clinical characteristics of the enrolled infants are demonstrated in Table 1. The study population included 592 preterm (33.7%) and 190 small for gestational age (SGA) (10.8%) infants. The median serum PIVKA-II levels measured using CLEIA were 42.0 (2.0, 29,501.0) mAU/mL. The overall incidence of PIVKA-II-positive cases (≥ 50 mAU/mL) was 42.8%, including 0.6% apparent VK deficiency (≥ 5000 mAU/mL), 3.1% experimental VK deficiency (1000–4999 mAU/mL), and 10.7% latent VK deficiency (200–999 mAU/mL) cases. A comparison of the characteristics of the preterm and term groups revealed that cesarean section and SGA were significantly more common (*p* < 0.001), while primiparity was significantly less common in the preterm group (*p* < 0.01). In contrast, serum PIVKA-II levels (term, 49.0 [2.0, 29,501.0] vs. preterm, 31.0 [8.0, 12,497.0] mAU/mL), the incidence of PIVKA-II-positive cases (49.4 vs. 29.7%, ≥ 50 mAU/mL), and VK deficiency (17.7 vs. 8.1%, ≥ 200 mAU/mL) were significantly higher in the term group (*p* < 0.001). In addition, the proportion of infants with different levels of high PIVKA-II concentrations was significantly different between the groups.

**Table 1:** Clinical characteristics of enrolled infants.

	Overall (n = 1759)	Preterm (n = 592)	Term (n = 1167)	*P*-value
Male (%)	928 (52.8)	308 (52.0)	620 (53.1)	0.70
Gestational age (weeks)	37.6 [22.4, 42.0]	34.6 [22.4, 36.9]	38.6 [37.0, 42.0]	< 0.001
Birth weight (g)	2710 [284, 4830]	2064 [284, 3732]	2952 [1586, 4830]	< 0.001
Primiparous	912 (51.8)	274 (46.3)	638 (54.7)	< 0.01
Cesarean section	1075 (61.1)	503 (85.0)	572 (49.0)	< 0.001
Apgar score at 5 min	8.8 ± 0.9	8.5 ± 1.2	8.9 ± 0.6	< 0.001
SGA	190 (10.8)	99 (16.7)	91 (7.8)	< 0.001
Range of PIVKA-II (mAU/mL)	42.0 [2.0, 29,501.0]	31.0 [8.0, 12,497.0]	49.0 [2.0, 29,501.0]	< 0.001
Number of PIVKA-II ≥ 5000 mAU/mL (%)	11 (0.6)	4 (0.7)	7 (0.6)	1.00
Number of PIVKA-II 1000–4999 mAU/mL (%)	54 (3.1)	10 (1.7)	44 (3.8)	0.02
Number of PIVKA-II 200–999 mAU/mL (%)	189 (10.7)	34 (5.7)	155 (13.3)	< 0.001
Number of PIVKA-II 50–199 mAU/mL (%)	499 (28.4)	128 (21.6)	371 (31.8)	< 0.001
Number of PIVKA-II < 50 mAU/mL (%)	1006 (57.2)	416 (70.3)	590 (50.6)	< 0.001

Data are expressed as number (%), mean ± SD, or median [range] as applicable. SGA is defined as a birth weight less than the 10th percentile of the mean value for Japanese newborns of the same gestational age. SGA, small for gestational age; PIVKA-II, protein induced by vitamin K absence or antagonist-II.

We also analyzed the follow-up values of PIVKA-II of the enrolled infants at 5 days after birth. The values were available for 1575 infants (89.5%). The clinical characteristics of these infants are presented in Table 2. They are almost consistent with those demonstrated in Table 1. As 5-day-old infants have routinely received prophylactic VK administration, the median serum PIVKA-II levels and the incidence of PIVKA-II-positive cases (≥ 50 mAU/mL, 16.4%), including apparent VK deficiency (≥ 5000 mAU/mL, 0.1%), experimental VK deficiency (1000–4999 mAU/mL, 0.6%), and latent VK deficiency (200–999 mAU/mL, 4.4%) at day 5, were lower than those at birth. Serum PIVKA-II levels (term, 21.0 [1.0, 7,933.0] vs. preterm, 15.0 [4.0, 29,242.0] mAU/mL), the incidence of PIVKA-II-positive cases (21.1 vs. 7.0%, ≥ 50 mAU/mL), and VK deficiency (6.5 vs. 2.5%, ≥ 200 mAU/mL) were significantly higher in the term group (*p* < 0.001). In addition, the proportion of infants with different levels of high PIVKA-II concentrations was significantly different between the two groups.

**Table 2:** Clinical characteristics of infants with the follow-up values of PIVKA-II at 5 days after birth.

	Overall (n = 1575)	Preterm (n = 525)	Term (n = 1050)	*P*-value
Male (%)	830 (52.7)	274 (52.2)	556 (53.0)	0.82
Gestational age (weeks)	37.6 [22.4, 41.9]	34.7 [22.4, 36.9]	38.6 [37.0, 41.9]	< 0.001
Birth weight (g)	2720 [284, 4830]	2072 [284, 3732]	2952 [1586, 4830]	< 0.001
Primiparous	815 (51.7)	241 (45.9)	574 (54.7)	0.001
Cesarean section	984 (62.5)	456 (86.9)	528 (50.3)	< 0.001
Apgar score at 5 min	8.8 ± 0.8	8.5 ± 1.1	8.9 ± 0.6	< 0.001
SGA	168 (10.7)	88 (16.8)	80 (7.6)	< 0.001
Range of PIVKA-II (mAU/mL)	19.0 [1.0, 29,242.0]	15.0 [4.0, 29,242.0]	21.0 [1.0, 7933.0]	< 0.001
Number of PIVKA-II ≥ 5000 mAU/mL (%)	2 (0.1)	1 (0.2)	1 (0.1)	1.00
Number of PIVKA-II 1000–4999 mAU/mL (%)	10 (0.6)	3 (0.6)	7 (0.7)	1.00
Number of PIVKA-II 200–999 mAU/mL (%)	69 (4.4)	9 (1.7)	60 (5.7)	< 0.001
Number of PIVKA-II 50–199 mAU/mL (%)	178 (11.3)	24 (4.6)	154 (14.7)	< 0.001
Number of PIVKA-II < 50 mAU/mL (%)	1316 (83.6)	488 (93.0)	828 (78.9)	< 0.001

Data are expressed as number (%), mean ± SD, or median [range] as applicable. SGA is defined as a birth weight less than the 10th percentile of the mean value for Japanese newborns of the same gestational age. SGA, small for gestational age; PIVKA-II, protein induced by vitamin K absence or antagonist-II.

Among all enrolled infants, only one was diagnosed with VK deficiency bleeding, with episodes of overt hematuria due to renal hemorrhage 2 days after birth. The case involved a female infant born as SGA at 33 weeks of gestation, with a birthweight of 1524 g (8.7 percentile of the mean value for Japanese newborns of the same gestational age^16^), and Apgar scores of 6 and 8 at 1 min and 5 min, respectively. Her mother (38 years old) underwent duodenojejunal bypass surgery for superior mesenteric artery syndrome at the age of 32 years and had anorexia due to lower back pain for 1 week before delivery. Hematuria was observed in the infant 6 h after birth, and left perinephric hematoma was noted on ultrasound at 1 and 2 days of age, resulting in a diagnosis of renal hemorrhage. In addition to prophylactic administration, two intravenous injections of vitamin K2 were administered, and the hematuria disappeared at 2 days of age. This infant’s PIVKA-II levels were 10,567 mAU/mL at birth and 2418 mAU/mL at day 5.

### Correlation between gestational age and PIVKA-II levels

**Figure 1:**
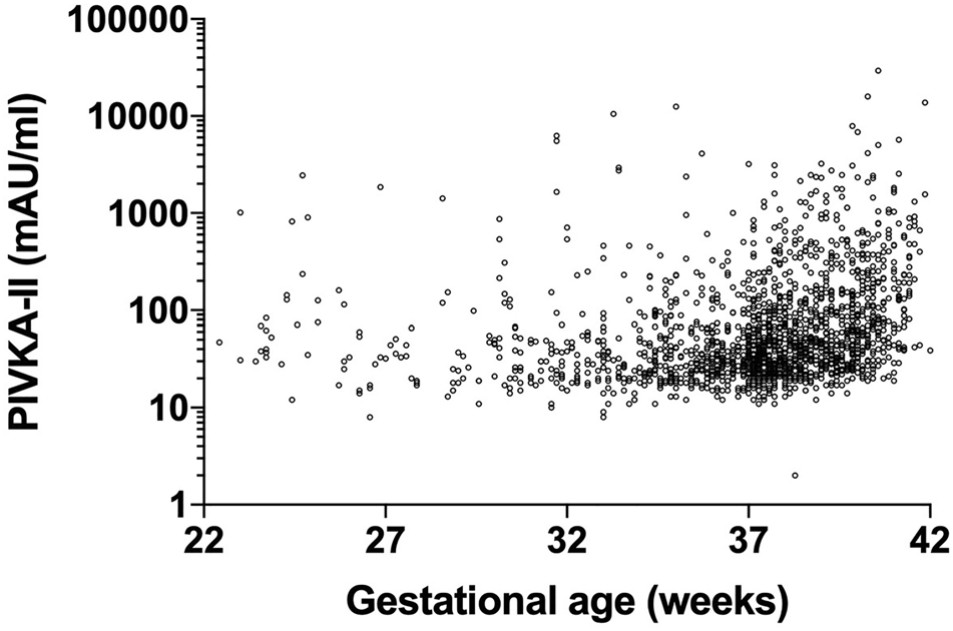
Correlation between gestational age and PIVKA-II levels. Gestational age correlates with PIVKA-II levels (mAU/mL, r^2^ = 0.117, *p* < 0.0001); PIVKA-II, protein induced by vitamin K absence or antagonist-II.

Gestational age correlated with PIVKA-II levels (r^2^ = 0.117, *p* < 0.0001, Fig. 1). We subsequently analyzed the correlation of PIVKA-II levels with gestational age between the preterm (< 37 gestational weeks, n = 592) and term (≥ 37 gestational weeks, n = 1167) groups. Interestingly, gestational age correlated with PIVKA-II levels in the term group (r^2^ = 0.135, *p* < 0.0001) but not in the preterm group (r^2^ = 0.001, *p* = 0.517).

## Discussion

In this study, we observed that the incidences of cases with detectable PIVKA-II concentrations in neonatal serum soon after birth were 0.6% for ≥ 5000 mAU/mL, 3.1% for 1000–4999 mAU/mL, 10.7% for 200–999 mAU/mL, and 28.4% for 50–199 mAU/mL. In addition, serum PIVKA-II levels after birth weakly correlated with gestational age.

A study conducted in the US in 1998 using cord blood obtained from 156 term infants recorded PIVKA-II levels of ≥ 100 mAU/mL in 47%, > 2000 mAU/mL in 7%, and > 10,000 mAU/mL in 2% of the infants^7^. In a 2006 UK study of cord blood samples from 90 preterm infants (< 32 weeks), 23% and 1% of the infants had PIVKA-II levels of ≥ 200 and ≥ 1000 mAU/mL, respectively^6^. A 2010 study conducted in Thailand using cord blood (683 cases, including 16 preterm infants) reported that 16%, 8.6%, and 1.5% of the cases had PIVKA-II levels of > 150, ≥ 1000, and > 5000 mAU/mL, respectively^3^. In a 2015 study from Uganda based on 141 cord blood samples (including 13 preterm infants), PIVKA-II levels of ≥ 200 and ≥ 5000 mAU/mL were noted in 66% and 22% of samples, respectively. In this study, 8/13 (61.5%) preterm infants also had PIVKA-II levels ≥ 200 AU/mL, and 3/13 (23%) had PIVKA-II levels ≥ 5000 mAU/mL^13^. A 2022 study from India that included 75 preterm infants with gestational age < 32 weeks reported that 49% of these infants had PIVKA-II levels > 28 mAU/mL, 2.7% had PIVKA-II levels ≥ 1000 mAU/mL, and none had PIVKA-II levels ≥ 5000 mAU/mL^14^. The strength of our study is that, to our knowledge, it enrolled the highest number of cases, including many preterm infants, as compared with previous reports. Our results are generally consistent with those of previous studies, except for a study from Uganda, which reported a substantially high incidence of abnormally high PIVKA-II levels. This difference observed in PIVKA-II levels between populations from Japan and Uganda (0.7% vs. 23% for ≥ 5000 mAU/mL) could be due to the differences in maternal nutritional status.

Serum PIVKA-II levels after birth were weakly correlated with gestational age. Although studies on PIVKA-II that include sufficient numbers of preterm infants are limited, Bovill et al. reported that the PIVKA-II positivity rate increased with increasing gestational age (5.3% at < 34 weeks, 5.7% at 34–38 weeks, and 9.9% at ≥ 38 weeks). However, the difference was not statistically significant^12^. Despite the small number of preterm cases, Santorino et al. reported that preterm birth was an independent risk factor for high PIVKA-II levels^13^. VK deficiency has been reported in 23% of preterm infants in the UK (n = 90)^6^ and 62% of preterm infants in Uganda (n = 13)^13^, which is substantially high compared to our study (8.1%, n = 592). However, the number of cases included in these studies was not sufficient. To our knowledge, our study is the first to measure PIVKA-II in neonates of all gestational ages and demonstrate that preterm infants are less prone to VK deficiency.

PIVKA-II levels in neonates can be influenced by external factors, such as VK deficiency, and internal factors, such as liver immaturity. We observed a significant correlation between gestational age and PIVKA-II levels in term infants but not in preterm infants. This suggests that some physiological changes in the intrauterine environment related to PIVKA-II production may occur around 37 weeks of gestation. According to a review comparing term pregnancy and prolonged pregnancy, microscopic changes, including aggregation of syncytiotrophoblast nuclei and reduced villous vascularity with concomitant impairment of trophoblast transport processes, were reported in placentas of prolonged pregnancy. These morphological changes are associated with increased evidence of oxidative stress, which could reflect placental aging or a reduction in placental function^17^. Whereas a study comparing oxidative stress markers between 37–39 weeks and ≥ 41 weeks placenta reported a significant increase in oxidative stress in the latter, suggesting placental aging or damage after 37 weeks’ gestation^18^. In addition, in a study examining the expression of DNA damage markers by gestational age in the normal placenta, immunohistochemical expression of 8-OHdG, an oxidative damage marker, significantly increased with gestational age^19^. Thus, we speculate that the aforementioned decrease in placental function after 37 weeks of gestation causes decreased placental transfer of VK to the fetus and contributes to physiological VK shortage in term newborns that was observed in this study. In a study evaluating the placental transfer of vitamin K1 using maternal, fetal, and neonatal blood samples, the mean maternal–fetal gradient of endogenous vitamin K1 concentrations at mid-trimester (14-fold) was lower than those at term (18-fold), which is consistent with our hypothesis^20^.

This study had several limitations. First, because this was a single-center retrospective study in Japan, it may not be applicable to other countries where the nutritional status and genetic background of pregnant women differ. Second, as data regarding maternal background and detailed neonatal information, except those examined in previous studies, were not available, care should be taken when interpreting the results of this study. Third, since we were not able to extract long-term clinical outcomes, we could not confirm the correlation between PIVKA-II level at birth and the risk of subsequent bleeding symptoms. Thus, this study alone could not clarify the PIVKA-II threshold for the risk of developing VK deficiency bleeding. Hence, future prospective studies that include detailed maternal and neonatal information are required.

## Conclusion

In Japan, the overall incidence of PIVKA-II-positive cases (≥ 50 mAU/mL) was 42.8%, including 0.6% apparent VK deficiency (≥ 5000 mAU/mL), 3.1% experimental VK deficiency (1000–4999 mAU/mL), and 10.7% latent VK deficiency (200–999 mAU/mL) cases. Serum PIVKA-II levels after birth were weakly correlated with gestational age, and VK deficiency was more commonly observed in term infants than in preterm infants.

## Methods

### Study design and patients

We retrospectively analyzed infants admitted to our hospital between June 1, 2018, and March 31, 2022, whose clinical and PIVKA-II data were available. To screen VK deficiency in neonates, we measured serum PIVKA-II levels at admission and 5 days after birth. According to the current Japanese guidelines^21^, the VK prophylaxis method in our hospital involves administering vitamin K2 (menatetrenone) within 24 h of birth (1 mg for infants with birthweight < 1500 g and 2 mg for infants with birthweight ≥ 1500 g) intravenously, if available, or orally. The second dose (2 mg) is administered at 4 days of age, intravenously or orally, in all cases^22,23^. Clinical data included gestational age, birth weight, sex, parity, delivery mode, Apgar scores, and SGA.

The study was conducted according to the guidelines of the Declaration of Helsinki and approved by the Ethics Committee at Kobe University Graduate School of Medicine (IRB approval number: B220244, March 29, 2023). All parents provided written informed consent for the use of their children’s personal medical data.

SGA was defined as having a birth weight less than the 10th percentile of the mean value for Japanese newborns of the same gestational age^16^.

We classified the patients into two groups according to their gestational age: preterm infants (< 37 gestational weeks) and term infants (≥ 37 gestational weeks). Clinical characteristics and serum PIVKA-II levels were compared between the two groups.

### Measuring PIVKA-II in the neonatal blood

Blood samples were collected within 2 h of admission and promptly centrifuged. Serum samples were stored at − 80 °C until use. With 20 µL of serum, we measured the PIVKA-II concentration using a two-step sandwich CLEIA with the LumipulsePresto PIVKAII-N kit (SEKISUI MEDICAL Inc., Japan). The data are expressed in arbitrary units, with 1 AU corresponding to 1 µg of purified prothrombin^24^.

### Statistical analysis

Data are presented as the median [range], mean ± SD, or number (%) as applicable. The Mann–Whitney nonparametric rank test, chi-square test, and Fisher’s exact test were used to compare data between the two groups as appropriate. Regression analysis was performed to linearly compare gestational age and serum PIVKA-II levels; regression equations and correlation coefficients (r^2^) were calculated. Statistical significance was set at *p* < 0.05. Analyses were performed using GraphPad Prism 7 software (GraphPad Software, Inc., La Jolla, CA, USA) and EZR (Saitama Medical Center, Jichi Medical University, Saitama, Japan), a graphical user interface for R 4.2.2 (R Foundation for Statistical Computing, Vienna, Austria).

## Data Availability

All data generated or analyzed during this study are included in this published article.
